# Comparison of Analgesic Effect Between Gabapentin and Diclofenac on Post-Operative Pain in Patients Undergoing Tonsillectomy

**DOI:** 10.5812/atr.7931

**Published:** 2012-10-14

**Authors:** Ahmad Yeganeh Mogadam, Mohammad Reza Fazel, Shohreh Parviz

**Affiliations:** 1 Trauma Research Center, Kashan University of Medical Sciences, Kashan, IR Iran; 2 Shahid Beheshti Hospital, Kashan University of Medical Sciences, Kashan, IR Iran

**Keywords:** Pain, Gabapentin, Diclofenac, Tonsillectomy

## Abstract

**Background:**

Tonsillectomy is a common procedure causing considerable postoperative pain. Postoperative pain intensity of 60 - 70 in the scale of visual analog scale (VAS) has been reported up to 3 - 4 days which could continue until 11 days after the surgery.

**Objectives:**

The current study aimed to compare the analgesic effect of gabapentin and diclofenac on pain after tonsillectomy with the control group.

**Patients and Methods:**

In this double-blind, placebo-controlled clinical trial, 90 patients aged 10-25 years, ASA classes I and II were randomly selected to receive 20 mg/kg oral gabapentin (n = 30), 1.0 mg / kg rectal diclofenac (n = 30) or placebo (n = 30) preoperatively. Pain was evaluated postoperatively on a visual analogue scale at 2, 6, 12 and 24 h. Opioid consumption in the first 24 h after surgery and the side effects were also recorded.

**Results:**

There was no significant difference in terms of age, sex, and time of surgery in the three groups. Patients in the gabapentin and diclofenac groups had significantly lower pain scores at all-time intervals than those in the placebo group. The total meperidine consumed in the gabapentin (14.16 ± 6.97 P = 0.001) and diclofenac (16.66 ± 8.95, P = 0.004) groups was significantly less than that of the placebo (33.4 ± 13.97) group. The frequency of side effects such as vomiting, dizziness, and headache was not significantly different among the groups.

**Conclusions:**

It can be concluded that gabapentin and diclofenac reduced postoperative pain and opioid consumption without obvious side effects.

## 1. Background

Postoperative pain is one of the most important and effective factors after surgery. Considering the type of surgery, different methods have been advised to control the pain. One of the most difficult pain management cases is related to larynx, especially tonsillectomy. Postoperative pain intensity of 60 - 70 in the scale of visual analog scale (VAS) has been reported up to 3 - 4 days which could continue until 11 days after the surgery (1). Pain after tonsillectomy can be due to different reasons such as tissue damage in the surgical site, postoperative edema, sensitization of peripheral nerves and central nervous system. Several factors are used to treat postoperative pain. Opioids are the most common medications for pain relief (2). However, they can put the patient at risk by high incidence of nausea and vomiting (3), leading to causing respiratory depression (4). Other widely used methods include non-steroidal anti-inflammatory drug (5), infiltration of local anesthetics or non-steroidal anti-inflammatory drugs, administration of prednisolone, applying neuroleptic drugs and blocking nerves (6). New evidence shows that using cyclooxygenase 2 inhibitors in appropriate doses can lead to pain reduction the same as non-steroidal anti-inflammatory drugs and can be used as alternatives for these drugs (7). Gabapentin is an anticonvulsant drug that probably applies its effects through voltage-dependent calcium channels (8). Gabapentin is applied to control chronic pains such as diabetic neuropathy (9), post-herpetic neuralgia (10) and other neuropathies (11). Recently, there have been several reports on postoperative pain control by gabapentin (12).

## 2. Objectives

This study aimed to compare the analgesic effect of gabapentin and diclofenac on pain after tonsillectomy with the control group.

## 3. Patients and Methods 

Ninety patients undergoing tonsillectomy aged 10 - 25 years old admitted to Matini hospital of Kashan University of Medical Sciences (KAUMS) during 2011 were enrolled to this double-blind, placebo-controlled clinical trial study. After obtaining the permission from ethics committee of the university and getting the informed written consent, table of random numbers was used to divide the patients to three groups of 30. The patients with the history of stomach bleeding, coagulation disorders, sensitivity to opioids and diclofenac, kidney diseases, asthma, chronic pains, epilepsy, diabetes, chronic use of analgesic and steroid drugs and drug addiction were excluded from the study. After admitting the patients to the surgery room, intravenous catheter was placed and their heart rate, blood pressure and arterial oxygen saturation were monitored. Thirty patients from the first group received 20 mg/kg gabapentin capsule one hour before the surgery. The second group received 1 mg/kg suppository diclofenac one hour before the surgery and the third group did not receive any medications. All the patients received 10 µg /kg alfentanil and 10 µg /kg atropine as premedication. After 3 - 5 minutes, anesthesia was inducted using thiopental 6 mg/kg, and 0.5 mg/kg atracurium was used to facilitate endotracheal intubation. The patients were ventilated by 100% oxygen and intubation with cuffed tubes was done in appropriate sizes. Anesthesia was continued using a mixture of oxygen and N2O with the ratio of 50% and isofloran. At the end of the surgery, content of the patient’s stomach was suctioned by a G-tube and its relaxant effect was reversed using neostigmine (70 µg /kg) and atropine (40 µg /kg). After extubation and ensuring adequate respiration, the patients were transferred to the recovery and were taken care of for two hours; then, they were transferred to the ward. Postoperative pain intensity was measured by visual analog scale at 2, 6, 12 and 24 h after the surgery and the starting time of feeding, nausea and vomiting, headache and dizziness were recorded. If the patients felt pain in the recovery room or in the ward, and if the pain score was more than 4, they received 25 mg intramuscular meperidine after recording the pain score. A sample size of 30 patients in each group was calculated to detect a significant difference of 15% (as derived from pilot data) in morphine consumption with a power of 85% and a significance level of 5%. SPSS statistical software was employed to analyze the data, student’s t-test and chi-squared test were used to assess significant differences between the two groups. Level of significance was P < 0.05.

## 4. Results

The results of this study showed that the two groups did not have any significant differences in terms of age, gender and duration of surgery (Table 1).

**Table 1:** Gender, Mean Age and Duration of Surgery in the Three Groups Under Study

	Gabapentin Group	Diclofenac Group	Placebo Group
**Age, Mean ± SD**	14.33 ± 2.55	14.8±2.7	14.65±2.6
**Gender (male/female)**	19.11	16.14	18.12
**Duration of surgery, Mean ± SD**	21.53±7.29	24.23±7.65	20.9±7.4

Differences of the mean of pain intensity at different hours between gabapentin and diclofenac groups were not significant (*P* > 0.05), patients’ pain in gabapentin group was significantly less than that of the placebo group (*P* < 0.05). The mean pain intensity in diclofenac group at 2 and 6 h after the surgery was significantly less than that of the placebo group (*P* < 0.05). This difference was not significant at 12 and 24 h (*P* > 0.05) (Table 2).

**Table 2:** Comparison of Pain Intensity in Three Groups Under Study

	Gabapentin Group, Mean ± SD	Diclofenac Group, Mean ± SD	Placebo Group, Mean ± SD
**2, h**	2.36 ± 1.52	2.56 ± 1.56	3.43 ± 1.34
**6, h**	3.56 ± 1.25	2.76 ± 1.43	2.1 ± 1.45
**12, h**	2.40 ± 1.16	1.96 ± 1.03	1.80 ± 1.06
**24, h**	2.23 ± 0.93	1.76 ± 0.97	1.53 ± 0.93

**Figure 1:**
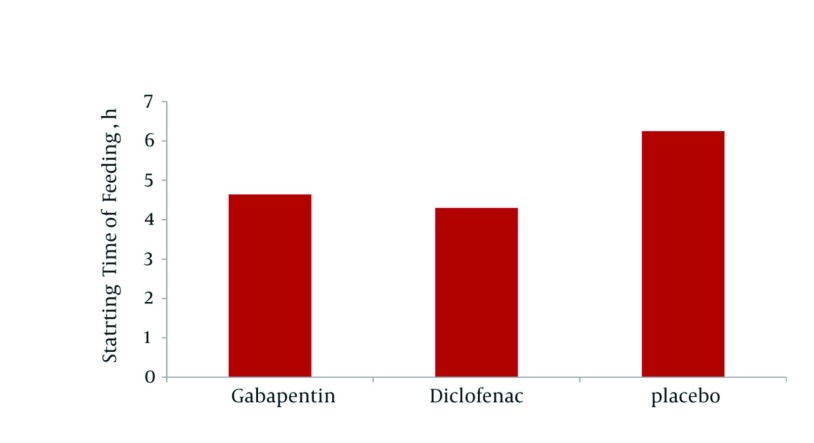
Mean Starting Time of Feeding in the Three Groups Under Study

Mean meperidine administered after the surgery was 14.16 ± 6.97, 16.66 ± 8.95 and 33.4 ± 13.97 mg in gabapentin, diclofenac and placebo groups, respectively. There was no significant difference in the amount of opioid consumption between the diclofenac and gabapentin groups (*P* = 0.59) but, it was significant between gabapentin and placebo (*P* = 0.001) and diclofenac and placebo (*P* = 0.004). Staring time of feeding after the surgery in gabapentin group (4.46 ± 1.13 h, *P* = 0.001) and diclofenac (4.3 ± 1.2 h, *P* = 0.001) was significantly less than that of the placebo group (6.26 ± 1.22 h); however, the two groups, Gabapentin and Diclofenac, were not significantly different (Figure 1).

The results of this study showed no significant difference between vomiting, headache and dizziness. (Table 3).

**Table 3:** Comparing Vomiting, Headache and Dizziness in the Three Groups Under Study

	Gabapentin Group, No.	Diclofenac Group, No.	Placebo Group, No.
**Vomiting**	6	7	11
**Dizziness**	20	22	24
**Headache**	13	10	11

## 5. Discussion

The results of this study demonstrated that administration of preoperative gabapentin and diclofenac significantly reduced postoperative pain and reduced the amount of opioid consumption. Thus far, no research has been done to compare the two drugs during tonsillectomy. Only in one study, the effects of these two drugs were compared after a sternotomy (13). Therefore, this study separately investigated the effect of these two drugs and opioid consumption. In a study conducted by Biyik *et al*., the effects of gabapentin and diclofenac on pain and opioid consumption after sternotomy were compared (13). The results showed the effectiveness of these two drugs on postoperative pain but, gabapentin had a longer effect. These results confirm the results of the current study. Here, only the effects of the two drugs were compared within 24 h but, in the above study, the patients used drugs for a long time which indicated the long term effect of gabapentin in comparison with diclofenac. In a study conducted by Mikkelsen *et al*. (6), the effects of gabapentin before and after surgery were compared with the placebo group. The patients received 1200 mg gabapentin before surgery, 1200 mg during 24 h after surgery and 1200 mg during 5 days after surgery. The results were similar to that of the current study; the amount of opioid consumption in the gabapentin group was significantly less than that of the placebo group. However, pain intensity was similar in the two groups, which was in contradiction with the present results. In Mikkelsen study, two groups of patients received 50 mg Refecoxib before and after the surgery. This is a non-steroidal anti-inflammatory drug which can have an effect on postoperative pain. Lack of differences in the two groups could be due to the consumption of this drug in the two groups. Similarly, administering diclofenac in the present study which is a non-steroidal anti-inflammatory drug can lead to lack of difference in the gabapentin and diclofenac groups. In a study conducted by Jeon *et al*. (14), patients in an experimental group was received 600 mg gabapentin before the surgery and the amount of opioid consumption and pain level was compared with those of the placebo group. The patients with postoperative pain received infusion through pump and IV diclofenac if required. The results of this study were similar to those of the present study which showed decreasing pain and opioid consumption after surgery. Oztekin *et al*. administered diclofenac suppository or placebo to the patients before tonsillectomy (15). In order to control pain after surgery, patients received morphine through infusion pump. The results of this study demonstrated less morphine consumption and less pain in the case group. These results confirmed the results of the present study. In another study conducted by Hiller *et al*. (16), the effects of diclofenac, paracetamol and their combination on pain after tonsillectomy were compared. Two drugs combination caused less pain after surgery but, there was no significant difference between the effects of diclofenac, and paracetamol. Some other studies also investigated the effect of diclofenac on pain after tonsillectomy surgery or other surgeries, all of which indicated the pain relieving effect of diclofenac and that less opioid consumption was required after surgery (17-19). These results confirmed the results of the current study. Side effects of gabapentin included vomiting, dizziness and headache (6). These side effects are seen in long term infusion of gabapentin or high doses of gabapentin administration. In a study conducted by Gilron *et al*. (6, 20) on 110 hysterectomy patients who took gabapentin and pain-relief non-steroid drugs and placebo, there was no significant difference in the levels of headache, dizziness and vomiting between the groups. In a study carried out by Jeon *et al*. (14), similar results were obtained in tonsillectomy. In this work, the side effects such as headache, dizziness and vomiting did not have any significant differences. Finally, this study showed that gabapentin and diclofenac administration can reduce pain and opioid consumption in comparison with the placebo group. Although this reduction level was higher in gabapentin group, the difference was not significant.
